# Preparation and Characterization of Preformed Polyelectrolyte and Polyampholyte Gel Particles for Plugging of High-Permeability Porous Media

**DOI:** 10.3390/gels10090562

**Published:** 2024-08-29

**Authors:** Gulnur Yelemessova, Iskander Gussenov, Aigerim Ayazbayeva, Alexey Shakhvorostov, Lyazzat Orazzhanova, Alexey Klivenko, Sarkyt Kudaibergenov

**Affiliations:** 1 Department of Chemistry and Ecology, Research School of Physical and Chemical Sciences, Shakarim University of Semey, Semey 071412, Kazakhstan; kussainova_g91@mail.ru (G.Y.); lyazzat.7070@mail.ru (L.O.); alexeyklivenko@mail.ru (A.K.); 2 Institute of Polymer Materials and Technology, Almaty 050019, Kazakhstan; iskander.gusenov@mail.ru (I.G.); ayazbayeva.aigerim@gmail.com (A.A.); skudai@mail.ru (S.K.); 3 Department of Chemical and Biochemical Engineering, Satbayev University, Almaty 050013, Kazakhstan

**Keywords:** PPG, hydrogel, swelling degree, polyelectrolyte hydrogel, polyampholyte hydrogel

## Abstract

Excessive reservoir water poses significant challenges in the oil and gas industry by diminishing hydrocarbon recovery efficiency and generating environmental and economic complications. Conventional polymer flooding techniques, although beneficial, often prove inadequate under conditions of elevated temperature and salinity, highlighting the need for more resilient materials. In this research, two types of acrylamide-based preformed particle gels (PPGs) were synthesized, as follows: polyelectrolyte and polyampholyte. These PPGs were engineered to improve plugging efficiency and endure extreme reservoir environments. The polyelectrolyte gels were synthesized using acrylamide (AAm) and sodium acrylate (SA), while the polyampholyte gels incorporated AAm, AMPS, and APTAC, with crosslinking achieved through MBAA. The swelling properties, modulated by temperature, salinity, and pH, were evaluated using the Ritger–Peppas and Yavari–Azizian models. The mechanical characteristics and surface morphology of the gels were analyzed using SEM and BET techniques. In sand pack experiments designed to mimic high-permeability reservoirs, the inclusion of 0.5 wt.% of fine PPGs substantially reduced water permeability, outperforming traditional hydrogels. Notably, the polyampholyte PPGs demonstrated superior resilience and efficacy in plugging. However, the experiments were limited by the low test temperature (25 °C) and brine salinity (26.6 g/L). Future investigations will aim to apply these PPGs in high-temperature, fractured carbonate reservoirs.

## 1. Introduction

The formation of excess reservoir water has become a significant issue in the oil industry, leading to a considerable amount of unrecoverable hydrocarbons remaining in the reservoir. As the oil reservoir ages, its heterogeneity increases, leading to the formation of an absorption zone in the high-permeability layer. Because of this heterogeneity and the presence of the absorption zone, the injected water tends to flow through the high-permeability layer, leaving a substantial amount of oil trapped in the low-permeability layers [1]. Excessive water extraction shortens the lifespan of wells [2,3], causes corrosion and scale deposits, and increases the load on surface infrastructure [4,5,6,7]. In many cases, high water production occurs because of the presence of thief zones, where the permeability can range from several to dozens of Darcies [8,9]

Generally, to address undesired water intake and enhance well production, various techniques known as conformance control are employed [10,11]. To achieve conformance during water flooding, a variety of materials are utilized, such as polymer injections, gel treatments, surfactants, foam filling, and other techniques [12,13,14].

But these methods have drawbacks, such as filtration issues with polymer injections, instability of gels, ineffectiveness of surfactants in high salinity.

To address the limitations of in situ gels, a different category known as preformed particle gels (PPGs) was developed for water shutoff and profile control. Unlike in situ gels, PPGs are polymerized into gels at surface facilities, then dried and ground to create PPG products [15].

One way to improve sweep efficiency and reduce water cut is by plugging of the high-permeability thief zones with a polymer gel [16]. In most cases, bulk polymer gels are used for this purpose. These gels are formed via a reaction between polyacrylamide and a crosslinker [17]. Polyacrylamide and its hydrolyzed derivatives are widely used in the petroleum industry due to their thickening ability [18]. This polymer is commonly used in the form of hydrolyzed polyacrylamide (HPAM) to achieve high viscosity within a specific range of brine salinity [19]. In fact, HPAM is the most widely used polymer today because of its low cost, availability, resistance to decomposition, and ability to increase viscosity [20]. For instance, 1 kg of HPAM can be purchased for approximately USD 2.5 [21]. However, plain HPAM gels fail to provide high resistance to the flow of brine in wide fractures. This is why the use of other materials, such as preformed gel particles, to achieve a higher degree of plugging has been extensively studied in recent years [2,22,23].

For instance, a recent review [24] suggests that, while PPGs’ characteristics are influenced by environmental factors, their overall structure remains stable under harsh conditions of high salinity (up to 200,000 ppm) and high temperature (up to 80 °C) for several months in most cases. Recent developments have demonstrated that sodium silicate and graphene nanoplatelets can enhance the temperature tolerance of PPGs [25]. The authors of [26] developed a method to create self-healing PPGs that can re-crosslink under extreme conditions. Gel particles ranging from 0.125 to 0.28 mm in size have been shown to reassemble at temperatures as high as 150 °C and in salt concentrations up to 20%. These advancements underscore the ongoing evolution of PPGs, making them highly promising for future applications in challenging reservoir environments.

Recently, numerous studies have focused on understanding the mechanism of PPG dispersion and evaluating the effectiveness of PPGs in highly permeable channels and fractures [27,28,29,30,31]. These studies have investigated the degree of swelling and mechanical strength of PPGs, focusing on the effects of particle size, PPG composition (e.g., concentration of monomer/polymer, crosslinking agent, and initiator) and reservoir conditions (including temperature, salinity, and pH). In addition, thermal stability and aging tests such as TGA and DSC have been carried out in other studies to ensure that PPGs can retain 80% of its original structure and strength at certain temperatures over an extended period of time [32]. Recent researches in this field are summarized in the Table 1.

As shown in the table, the existing PPG systems are significantly affected by the environmental solution factors, influencing their degree of swelling. This limitation restricts their application to some extent. The use of polyampholytic hydrogels as PPGs addresses this issue, allowing the hydrogels to swell independently of environmental factors such as ionic strength and pH. This greatly expands their potential range of applications.

A review of the literature indicates that most reported nano- and microgel possess nonionic, anionic, or cationic behavior. Polyampholytic nano- and microgels are particularly intriguing due to their responsiveness to a range of external stimuli, including temperature, pH, salt composition, solvents, electric or magnetic fields, and light radiation. These gels are significant potential for developing “smart” materials applicable in medicine, biotechnology, nanotechnology, catalysis, the oil industry, environmental protection, and beyond [38,39,40]

Ma et al. [41] in their recent review highlight the five main research directions for PPGs currently being explored, as follows:

1. High-Temperature-Resistant PPGs;
2. Re-Crosslinkable PPGs;
3. Delayed-Swelling PPGs;
4. Augmented PPGs;
5. Degradable PPG.

A series of low-charge-density amphoteric terpolymers based on acrylamide (AAm), 2-acrylamido-2-methyl-1-propanesulfonic acid sodium salt (AMPS), and (3-acrylamidopropyl) trimethylammonium chloride (APTAC) were developed and their viscosifying ability with respect to reservoir saline water (200–300 g∙L^−1^) at 60 °C were evaluated [20,42,43,44,45]. These terpolymers exhibit thermal stability and can swell and increase viscosity in salt water due to a specific “antipolyelectrolyte” effect [44]. Polyampholytes are charged macromolecules that carry both anionic and cationic groups along the polymer chain [46]. They can be synthesized through classical and controlled free radical polymerization, anionic polymerization, and group transfer polymerization (GTP). The behavior of polyampholytes in aqueous solution is influenced by Coulomb interactions between the basic/cationic and acidic/anionic residues. Polyampholytes exhibit both polyelectrolyte and antipolyelectrolyte behavior in aqueous and aqueous-salt media. Critical parameters influencing their behavior include charge density, charge asymmetry (the degree of charge balance), the distance between charges and their distribution, the charge of the substrate surface, structural conformation, and the ionic strength of the solution. Polyampholytes are of great interest because of their versatility and are used in various technological processes such as water purification, enhanced oil recovery (EOR), sludge dewatering, paper production, pigment retention, mineral processing, and flocculation [47].

The aim of our research was to develop a new type of polyampholyte PPG with enhanced performance compared to conventional and widely recognized polyelectrolyte PPGs. For this purpose a series of polyelectrolyte and polyampholyte hydrogels based on acrylamide derivatives were synthesized and studied as plugging agents for high-permeability channels in oil reservoirs.

## 2. Results and Discussion

**Figure 1:**
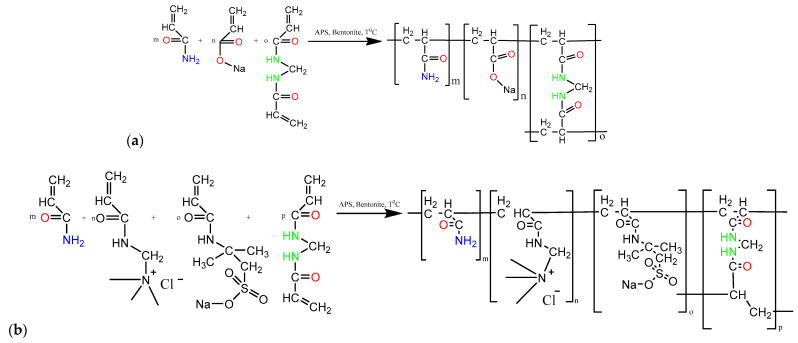
Synthetic protocol of polyelectrolyte (**a**) AAm-SA and polyampholyte (**b**) AAm-APTAC-AMPS hydrogels.

Polyelectrolyte hydrogel of the composition [AAm]:[SA] = 95:5 mol.% (AAm_95_-SA_5_) (Figure 1a) and polyampholyte hydrogel composed of [AAm]:[APTAC]:[AMPS] = 95:2.5:2.5 mol.% (AAm_95_-APTAC_2.5_-AMPS_2.5_) (Figure 1b) were synthesized by free radical copolymerization as described in [48,49]. The selection of component ratios for PPG synthesis has been thoroughly investigated in our previously published works [48,49]. The selection of specific monomers from the wide range of commercially available options is based on the fact that the chosen monomers (acrylamide and sodium acrylate) are cost-effective, readily available in large quantities, and are widely used at present. The choice of monomers, AMPS and APTAC, for synthesis of polyampholytic PPGs is driven by their ease of participation in free radical polymerization with high yield and their availability in large volumes. Additionally, our research group has extensive experience in utilizing these monomers for the synthesis of functional polymers for bionanotechnology, environmental science, catalysis, and oil recovery [48,49]. The impact of monomer concentrations on the properties of the resulting hydrogels has been thoroughly studied in our previously published works [48,49]. In this study, the following combinations of components were used: AAm/SA (95/5) and AAm/APTAC/AMPS (95/2.5/2.5). The selection of the relative concentrations of monomers (see Section 2, Materials and Methods) is based on the fact that these specific combinations allow for the production of hydrogels with the desired properties, namely, swelling capacity, mechanical strength, and resistance to harsh environmental conditions (pH, temperature, and high salinity).

**Figure 2:**
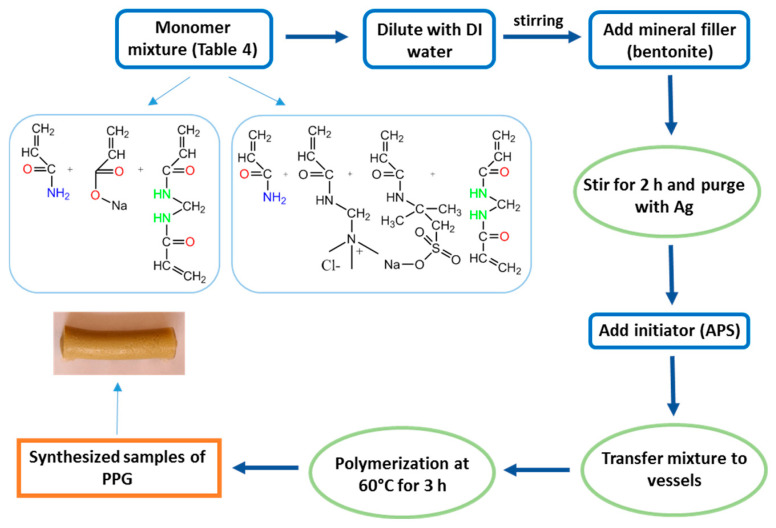
Hydrogel synthesis process.

A flow chart of the synthesis process of hydrogels, with each step identified and arranged in a logical sequence, is shown in Figure 2.

The yield of the products was determined gravimetrically by weighing the mass of the obtained hydrogel after washing it in distilled water and thoroughly drying it, relative to the mass of the initial reagents, and was found to be 91.21 ± 0.90% for AAm_95_-SA_5_ and 88.33 ± 1.38% for AAm_95_-APTAC_2.5_-AMPS_2.5_.

### 2.1. FTIR Spectroscopy Results

**Figure 3:**
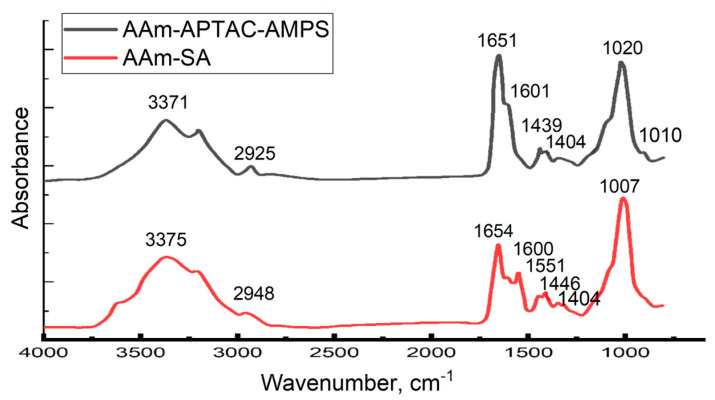
FTIR spectra of hydrogels: Am_95_-SA_5_; AAm_95_-APTAC_2.5_-AMPS_2.5_.

Figure 3 shows the FTIR spectra of AAm_95_-SA_5_ and AAm_95_-APTAC_2.5_-AMPS_2.5_ hydrogels.

The peaks at 3371–3375 cm^−1^ correspond to the stretching vibrations of the N-H bond of acrylamide. The absorption bands in the range of 2925–2948 cm^−1^ are attributed to the asymmetric and symmetric vibrations of CH groups. The bands at 1651–1654 cm^−1^ are associated with the vibrations of N-substituted amide I groups, while peaks at 1601–1600 cm^−1^ correspond to the vibrations of N-substituted amide II groups. The peak at 1551 cm^−1^ belongs to the vibrations carboxylate units (COO^−^) of acrylic acid. The absorption band at 1439–1446 cm^−1^ corresponds to the stretching vibrations of CN groups, and the band at 1404 cm^−1^ is characteristic for the stretching vibrations of CH groups. The peak at 1007–1020 cm^−1^ indicates the stretching vibrations of Si-O in bentonite. Thus, the FTIR spectra of composite hydrogels show the characteristic bands of carboxyl, hydroxyl, and methylene groups, and the absence of double bonds demonstrates that the polymerization process is fully completed. The specific reference group for the APTAC is a quaternary ammonium species (-N+(CH3)3), which does not have a clear, even absorption band, have only a small intensity peak at 950 cm^−1^. For the AMPS monomer-specific reference group could be a sulfonates groups (-RSO3-) that have absorption peak generally at 1080–1010 cm^−1^. These small differences in the FTIR spectra between Aam-SA and Aam-APTAC-AMPS could be seen in the 1100–950 cm^−1^ region, which is strongly overlapped by a very intensive Si-O-Si absorption band.

### 2.2. Swelling Kinetics of Hydrogels Determined by Using Ritger–Peppas and Yavari–Azizyan Models

Since hydrogels can retain water due to their three-dimensional polymer mesh, their swelling ability and swelling rate depend on various parameters, such as temperature, pH, ionic strength, degree of crosslinking, and type of monomers. Various kinetic models of the swelling rate have been presented, but most of them describe either diffusion or relaxation of the polymer as the predominant process affecting the swelling rate.

The swelling kinetics of hydrogel in water have been studied using a gravimetric approach [50]. The equilibrium swelling degrees are reached after approximately 50 h of equilibration. To understand the mechanism of water sorption by hydrogels, the kinetic data were analyzed using a semi-empirical model proposed by Ritger and Peppas [51] (Equation (1)).

(1) $$\frac{{SD}_{t}}{{SD}_{\infty }}=k\cdot t^{n}$$

where ${SD}_{t}—$swelling degree (g·g^−1^) at time t (min), ${SD}_{\infty }—$swelling degree at equilibrium (g·g^−1^), *k*—is a characteristic constant of a hydrogel, and *n*—is a characteristic exponent of the transport mode of the penetrant (water).

It should be noted that this equation is valid only for *SD_t_*/*SD*_∞_ ≤ 0.6. The value of *n* provides information about the mechanism of water sorption. When 0 ≤ *n* ≤ 0.5, the sorption of water by the samples follows Fick’s law, indicating that diffusion is the rate-limiting factor and much slower than the relaxation rate of polymer chains. When 0.5 < *n* < 1.0, it indicates a deviation from Fick’s law, suggesting that water absorption is controlled by both diffusion and relaxation of polymer chains. If *n* ˃ 1, then the absorption of water is mainly controlled by the relaxation of chains, meaning the rate of water diffusion is greater than the rate of polymer chain relaxation.

**Figure 4:**
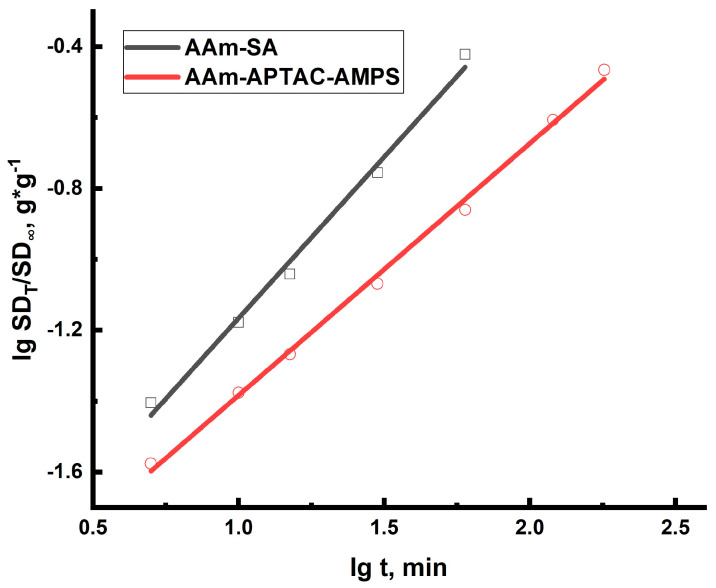
Graphical description of the kinetics of hydrogel swelling according to the Rittger–Peppas model [52].

The experimental results obtained in this study were analyzed by plotting the data in lg(*SD_t_*/*SD*_∞_) − lg(*t*), coordinates to obtain a linear correlation at *SD_t_*/*SD*_∞_ < 0.6. The values of *n* were determined from the slope of the graph. The results are shown in Figure 4 and listed in Table 2.

The swelling kinetics of hydrogel particles were described using the method of Yavari and Azizyan [52]. Unlike the Ritger and Peppas model, this model allows for the description of the swelling process over the entire range of *SD* and determines whether the process is dominated by diffusion or relaxation, using Equation (2), as follows:

(2) $$S_{t}=S_{e}\left[1-e^{\left(-k_{1}t-k_{2}t^{1/2}\right)}\right]$$

where $S_{t}$—swelling of hydrogel (g·g^−1^) at time t; $S_{e}$—equilibrium swelling of hydrogel (g·g^−1^); and $k_{1}\;\mathrm{and}\;k_{2}$—rate of swelling constants.

**Figure 5:**
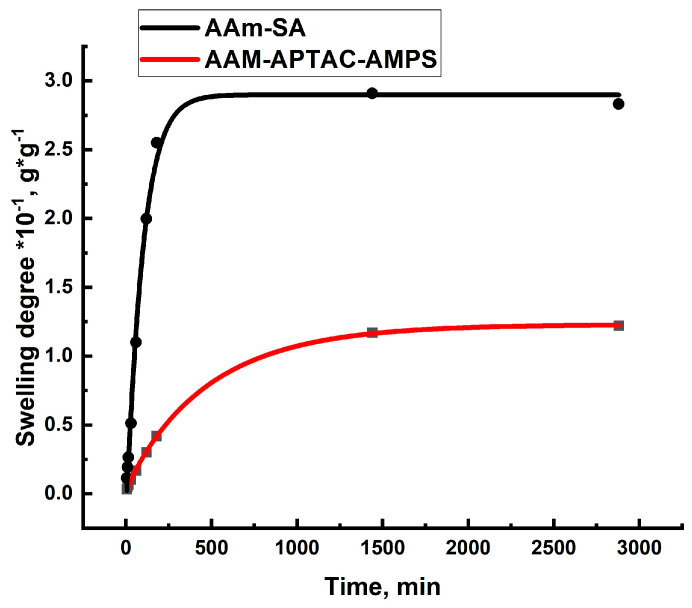
Swelling kinetics of hydrogels in distilled water.

Figure 5 shows the swelling kinetics of polyelectrolyte and polyampholyte hydrogels in distilled water obtained according to Equation (2).

As can be seen from Figure 4, the swelling rate of the AAm-SA hydrogel was much higher than the AAM-APTAC-AMPS. In the case of the polyampholytic hydrogels, the swelling rate in distilled water was lower due to the competition between two factors—the dissociation of charged units under the influence of the solvent and the formation of intramacromolecular bonds between oppositely charged units of the polyampholytic hydrogel [53]. The AAm-SA hydrogel cannot form intramacromolecular bonds, which is why it swells significantly faster and to a greater extent. It is clearly seen that the points on the curve are positioned with high accuracy (R^2^ = 0.99), indicating a well-constructed model. If *k*_1_ ≫ *k*_2_, then the swelling rate of the gels is determined by the relaxation of polymer chains. Conversely, if *k*_1_≪*k*_2_, the swelling rate is determined by diffusion. If the values of *k*_1_ and *k*_2_ are close to each other, the swelling rate is influenced by both diffusion and relaxation processes. Table 3 summarizes the swelling kinetics of the two types of hydrogels. The data indicate that for hydrogels AAm_95_-SA_5_ and AAm_95_-APTAC_2.5_-AMPS_2.5_ the swelling process mechanism corresponds to both diffusion and relaxation processes.

As evidenced from Table 1 and Table 2, both models exhibit a similar mechanism for fluid transport within the gel matrix. This finding generally aligns with the understanding that the Azizyan model provides a broader framework. Under satisfactory conditions, it describes the gel swelling behavior in a comparable manner. The Rittger and Peppas model, being more simplified, does not require specialized mathematical models for plotting and adequately describes the swelling of gels at swelling degrees not exceeding 0.6.

Anomalous transport, indicated by the *n* values for both hydrogels, suggests that the diffusion mechanism deviates from classical Fickian diffusion [51]. In the context of hydrogels used as plugging agents, this non-Fickian behavior can significantly impact their performance. Non-Fickian transport may lead to a more gradual and prolonged swelling process. This can enhance the gel’s ability to conform to the irregularities and heterogeneities within the high-permeability channels. The slower and more controlled swelling might allow the hydrogel to fill the pore spaces more completely and uniformly, improving the seal and reducing the risk of channel reopening. Finally, non-Fickian behavior indicate that system can save its integrity under varying reservoir conditions (temperature, pH, etc.).

### 2.3. Temperature-, Salt-, and pH-Dependent Swelling of PPGs

**Figure 6:**
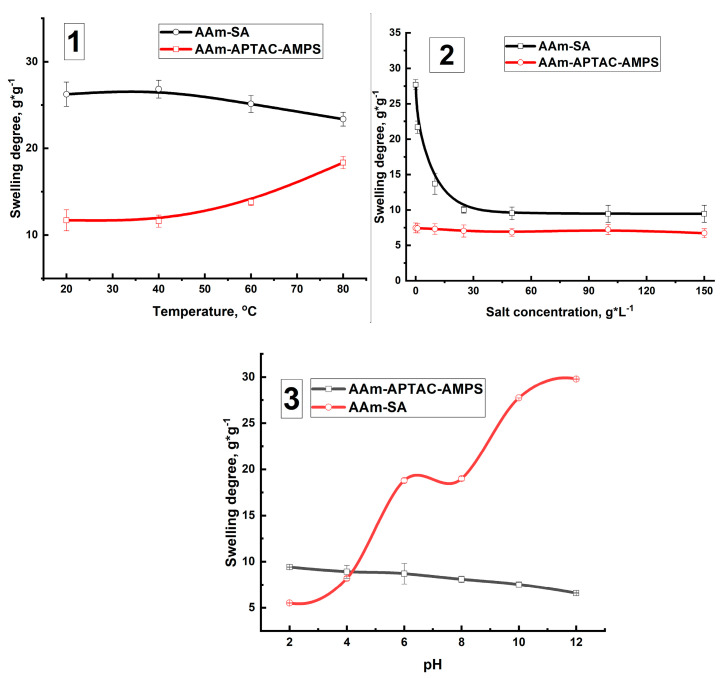
Dependence of hydrogel SD on temperature (**1**), ionic strength (**2**), and pH (**3**).

Swelling is the primary parameter that determines the suitability of a hydrogel as a PPG. The swelling degrees of polyelectrolyte and polyampholyte hydrogels in dependence on temperature, pH, and salt addition were compared (Figure 6).

As shown in Figure 6(1), the SD of hydrogels does not significantly depend on ambient temperature within the range of 20 to 80 °C. This observation aligns with previous findings by other researchers [54]. The stability of the studied systems under high-temperature conditions is evidenced by the DSC and TGA results published in our previous works [48,49]. It has been established that the studied systems remain stable up to a temperature of 150 °C. The SD of AAm_95_-SA_5_ hydrogel was notably higher, reaching up to 26 g∙g^−1^. This elevated SD is attributed to the presence of sodium acrylate groups, which expand thereby stretching the polymer network. In contrast, the AAm_95_-APTAC_2.5_-AMPS_2.5_ hydrogel exhibited a lower SD of approximately 18 g∙g^−1^. This reduced swelling was due to the electrostatic attraction between the oppositely charged monomers within the polymer matrix [55].

PPGs are utilized in formation water conditions, which can exhibit salinity levels ranging from a few grams per liter (g∙L^−1^) to several hundred g∙L^−1^, depending on the type of oil field. Figure 6(2) illustrates the relationship between the SD of hydrogels and salt concentration. The addition of sodium chloride (NaCl) leads to a significant decrease in the SD of AAm_95_-SA_5_ hydrogel from 25 g∙g^−1^to 10 g∙g^−1^. This decrease is attributed to the suppression of the polyelectrolyte effect at high ionic strength, which results from the equalization of osmotic pressure between the hydrogel and the surrounding solution. The presence of ions in the solution reduces the osmotic pressure gradient, which is the primary driving force for the swelling of the hydrogel, thereby decreasing the extent of swelling [56,57].

For AAm_95_-APTAC_2.5_-AMPS_2.5_ hydrogel, the swelling degree (SD) is not influenced by the salt concentration due to the quasi-electroneutral state of macromolecular chains, resulting from the electrostatic attraction between oppositely charged segments [20,58,59] (Figure 6(2)).

The SD of the hydrogels was also measured in formation water from the Karazhanbas field, with an average salinity of 26.6 g∙L^−1^. The SD of the hydrogels determined in model brine and real saline water coincides well.

Studying the effect of pH on the SD is crucial because the synthesized PPG contains functional groups whose degree of dissociation significantly can depend on the pH of the surrounding environment. Figure 6(3) illustrates the dependence of SD on pH. The SD of AAm_95_-SA_5_ hydrogel gradually increases from 6 to 30 g∙g^−1^ as the pH ranges from 2 to 12. At low pH, the ionization of carboxylate ions (COO⁻) is suppressed, and the COO⁻Na⁺ groups convert to their undissociated state (COOH), leading to hydrogel shrinking [60]. At high pH, the carboxylate ions remain ionized, enhancing the polyelectrolyte effect, which results in more pronounced swelling of the hydrogel [61].

The SD of amphoteric hydrogel AAm_95_-APTAC_2.5_-AMPS_2.5_ remains unchanged, staying around 10 g∙g^−1^. This is because the AAm_95_-APTAC_2.5_-AMPS_2.5_ belongs to quenched (or fully charged) polyampholyte consisting of static positive and negative charges that slightly depend on pH [62,63].

### 2.4. Study of the Mechanical Properties of Hydrogels

The results of the mechanical analysis of hydrogels are the following: the Young’s modulus of the AAm_95_-SA_5_ hydrogel is 85.13 ± 0.36 Pa, and for the AAm_95_-APTAC_2.5_-AMPS_2.5_ hydrogel, it is 91.01 ± 0.4 Pa. So, the strength of the hydrogels is suitable for use as a PPG [56]. Strain–stress curves and dependence of Young moduli on the hydrogel composition of studied hydrogels are presented in the Figures S1 and S2.

### 2.5. SEM Images and Porosity of Hydrogels

**Figure 7:**
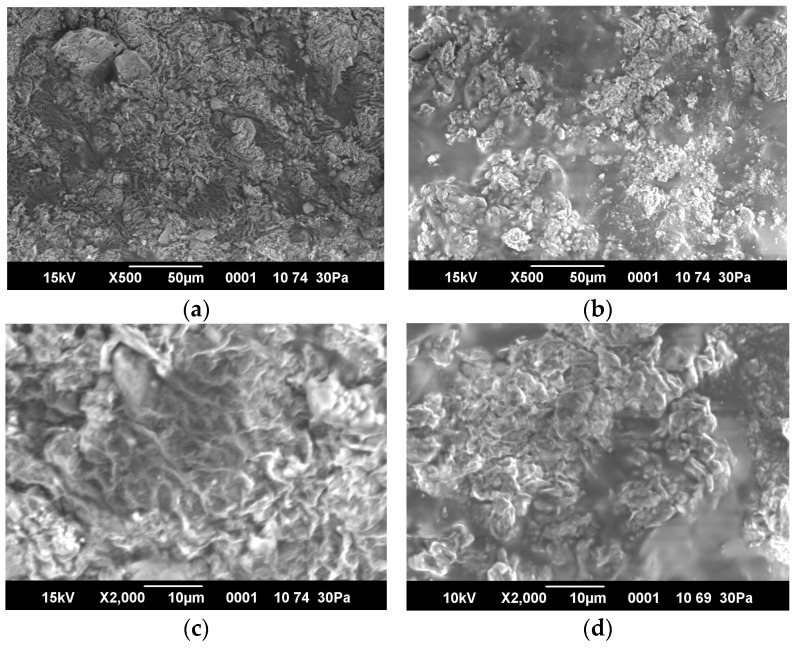

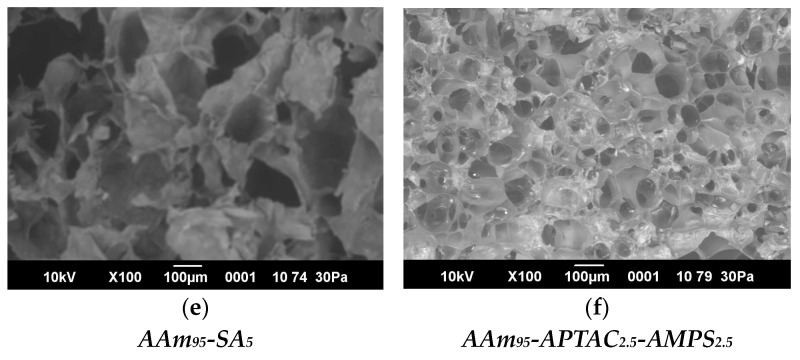
SEM images of hydrogel samples. (**a**–**d**) samples without freeze drying, (**e**,**f**)—freeze dried samples.

Evaluation of the morphology of hydrogels is important from practical point of view. If pores are present, the hydrogels can allow liquid to pass through them, which is a negative factor in terms of increasing reservoir pressure. Figure 7 shows the morphology of hydrogels examined with a low-vacuum analytical scanning electron microscope.

Figure 7a–c, and d show that the hydrogels do not have pores. Pores are not visible even at 2000× magnification. However, if swollen hydrogels subjected to critical freezing are observed under a microscope, pores can be detected (Figure 7e,f), which appear due to the release of ice crystals in the hydrogel. Thus, it can be stated that the hydrogels do not have pores. This is also confirmed by the results of BET analysis.

The weight loss of the AAm_95_-SA_5_ and AAm_95_-APTAC_2.5_-AMPS_2.5_ hydrogel samples during the pretreatment at 80 °C in a vacuum before the krypton adsorption was 2.4 m∙m%^−1^ and 4.5 m∙m%^−1^ for respectively. The mass loss is an indication of the desorption of the adsorbed gases/components from the surface of the sample. Both samples are nonporous materials with low surface area roughly estimated, as follows:

The AAm_95_-SA_5_ single point surface at p/p_0_ = 0.2490 relative pressure is 0.012 m^2^∙g^−1^. The AAm_95_-APTAC_2.5_-AMPS_2.5_ single point surface area at p/p_0_ = 0.2979 relative pressure is 0.006 m^2^∙g^−1^. Mainly the geometry of the pellets determines the external surface area of the investigated samples. No nitrogen low temperature adsorption was detected for hydrogel samples.

### 2.6. Sand Pack Flooding

#### 2.6.1. Darcy Sand Pack Flooding

In the first experiments plain HPAM gelling solutions were injected into the 5.5 Darcy sand pack model in order to determine the permeability reduction inside of unconsolidated sand without discrete high permeability conduits. The established practice is to use 0.5 wt.% 6–7 mln Da HPAM/0.05 wt.% chromium acetate; however, taking into account long gelation time of this recipe at low reservoir temperature (over several days), the concentration of the crosslinker in this experiment was increased to 0.5 wt.% which provided moderately flowing gel in one day of aging at 25 °C (gel D according to Sydansk gel strength code). With the further aging over 2 weeks the gel improved its strength up to gel D with no signs of syneresis.

The oil-saturated and water-flooded sand pack model was subjected to an injection of around 3 PVs of 0.5 wt.% 6–7 mln Da HPAM/0.5 wt.% chromium acetate gelling solution (Figure S3).

**Figure 8:**
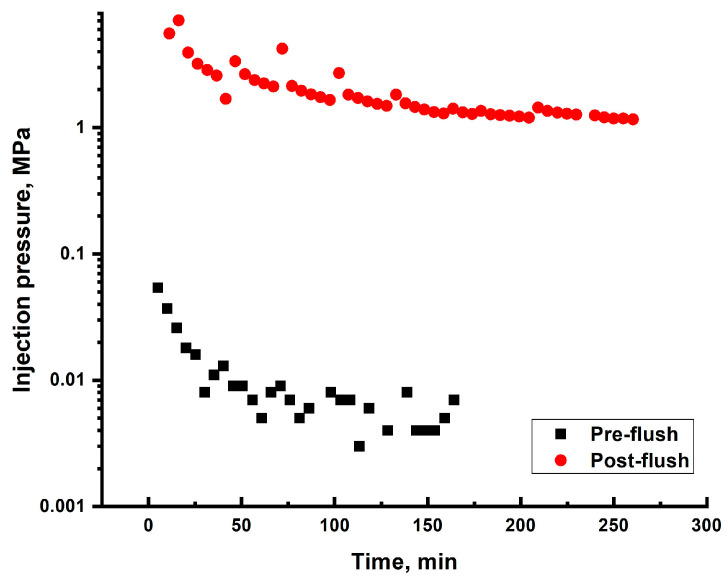
Comparison between pre-flush and post-flush pressure values.

After the injection of gellant the model was aged for 3 days and subjected for post-flush brine injection. Figure 8 compares pre-flush and post-flush injection pressure values. As can be seen from the graph, after the gel polymer treatment of the model, the water injection pressure increased approximately 190 times (from 0.006 to 1.12 MPa). Considering that the water filtration before and after the gel polymer treatment was conducted under equal conditions (flow rate and temperature), it is reasonable to establish a 190-fold reduction in water permeability.

**Figure 9:**
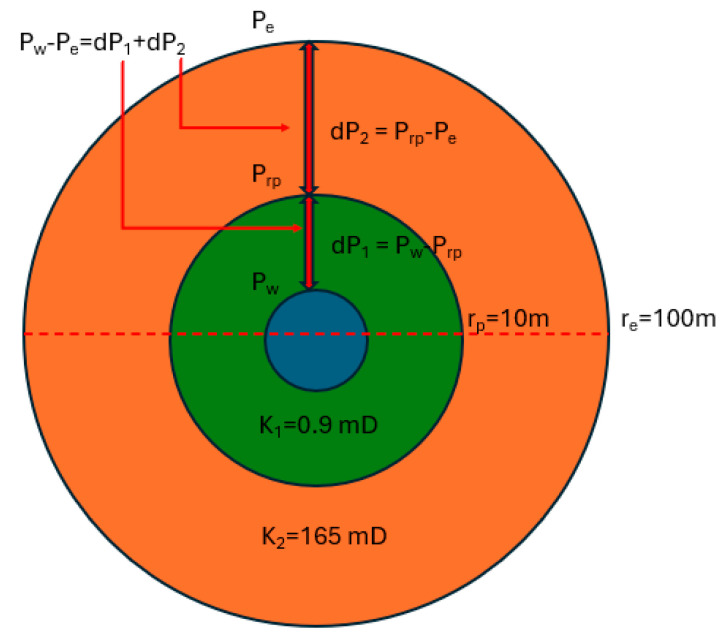
Radial flow model in a homogeneous reservoir.

Simple calculations by using Darcy flow equation (Equation (3) and Figure 9) show that if the penetration distance of the gelling solution is just 1 m, the injectivity of the well may decrease by 60 times.

(3) $$\frac{q\mu ln\frac{r_{e}}{r_{w}}}{2\pi hk_{av}}=\frac{q\mu ln\frac{r_{p}}{r_{w}}}{2\pi hk_{1}}+\frac{q\mu ln\frac{r_{e}}{r_{p}}}{2\pi hk_{2}},$$

If the well intersects two layers with different permeabilities, the ratio of the volumes of the gel polymer composition that penetrates the first and second layers can be found using Equation (4), as follows:

(4) q1q2=2πk1h1dPμlnrerw2πk2h2dPμlnrerw=πr12h1φ1tπr22h2φ2t=k1k2=r12φ1r22φ2,

For example, if 5 m^3^ of the gel polymer composition was injected into the well, with a permeability contrast of *k*_1_/*k*_2_ = 10, then 4.55 m^3^ will penetrate the more permeable layer, and 0.45 m^3^ will penetrate the less permeable layer. This corresponds to penetration distances of 1.2 m and 0.38 m, respectively, given a layer thickness of 5 m and a porosity of 20%. With a permeability reduction factor of 190, the average permeability and, hence, the injectivity of the two layers will decrease by 69 and 38 times, respectively.

Thus, it is evident that if there are no high-permeability channels in the near-wellbore zone, the injection of the gel polymer composition into unconsolidated sandstone, whether in a homogeneous or layered bed, will lead to a significant reduction in injectivity. However, in practice, this rarely occurs because gel polymer compositions penetrate high-permeability channels and/or fractures. The presence of such channels complicates the process of oil displacement by water.

Thus, using multi-Darcy sand packs with large pores is more practical for assessing the reduction in water permeability caused by PPGs and plain polymer gels. The following section describes the results obtained using plain and PPG-impregnated HPAM gels.

#### 2.6.2. >60 Darcy Sand Pack Flooding

The first experiment was conducted by using a commonly known polymer gel recipe containing 0.5 wt.% of 6–7 mln Da HPAM with 5% hydrolysis degree. The concentration of chromium acetate was increased up to 0.2 wt.% in order to accelerate gelation at 25 °C. 1 PV of this gelant was injected into the 8.6-cm-long sand pack model at pressure gradients which were not detectable by using pressure transducers with minimal sensitivity limit of +/− 0.004 MPa. The model was aged for 2 days to achieve moderately flowing state of the gel (gel D in Sydansk gel strength code). The following post-flush in the highest injection pressure value of 0.016 MPa corresponding to the pressure gradient of 0.2 MPa/m. The results of this experiment can be used for the comparison, as [0.5 wt% of 6–7 mln Da HPAM with 5% hydrolysis degree/chromium acetate] is one of the most widely used polymer gel formulations.

**Figure 10:**
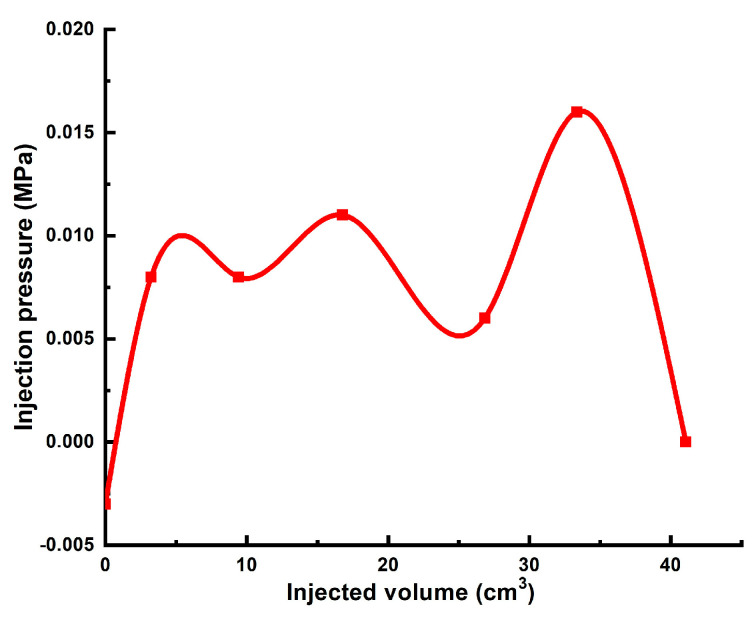
Injection pressure vs. injected volume during the post-flush after treating 60 Darcy sand pack with plain 0.5 wt.% HPAM gel. Flow rate—0.5 cm^3^∙min^−1;^, temperature—25 °C.

**Figure 11:**
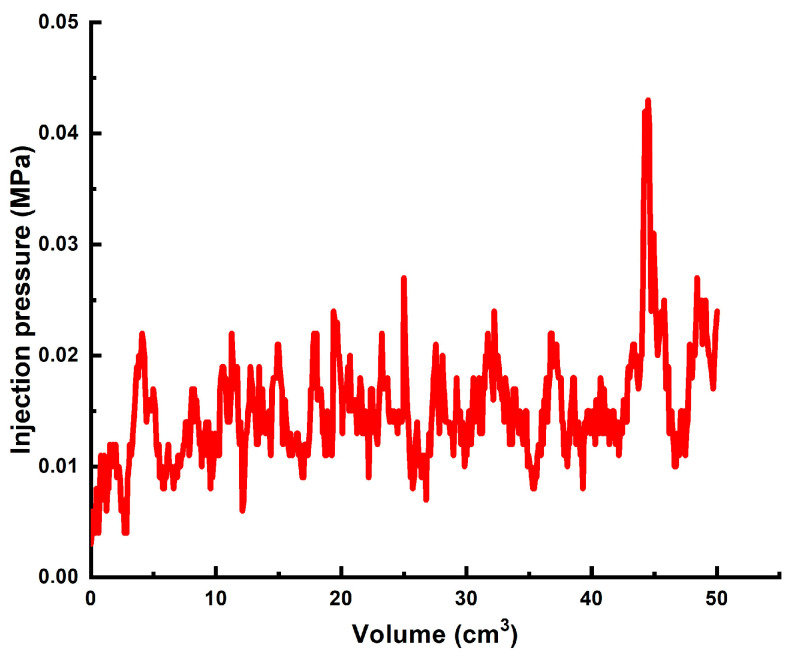
The injection pressure vs. injected volume registered during the injection of mature [0.5 wt.% HPAM/0.5 wt.% coarse PPG/0.05 wt.% chromium acetate] gel into the sand pack model.

In order to achieve a better result, in the next experiment polymer gel was impregnated with preformed partial gel particles. Figure 10 demonstrates the injection pressure documented during the injection of mature [0.5 wt.% 13–18 mln Da and 16% hydrolysis degree HPAM/0.5 wt.% coarse (1–2.5 mm) PPG/0.05 wt.% chromium (III) acetate] gel (Figure 11 into the high permeability sand pack. Of note is that the gel injection was started 24 h after the addition of the crosslinker. The pressure fluctuations are explained by the temporary gel screen outs.

After the gel injection the model was aged for 1 day and subjected to the post flush at 1 cm^3^/min. The highest injection pressure recorded during the brine injection into the model was 0.006 MPa, which is close to the sensitivity limit (+/− 0.004 MPa) of the pressure measuring device used in this study. This low post-flush injection pressure is due to the fact that most of the PPG particles could not reach the model or penetrate the porous media. In fact, most PPGs were retained inside the piston accumulator, and those that passed through the 4 mm diameter injection line were retained at the face of the sand pack model (Figure S4a). Given the relatively large cross-sectional area of the pack (14.5 cm^2^), the water injected during the post-flush phase easily flowed through the gel, bypassing the retained PPGs.

Given that the PPGs could not penetrate the >60-Darcy sand pack model (Figure S4b), this experiment was considered equivalent to using plain gel for the baseline comparison.

**Figure 12:**
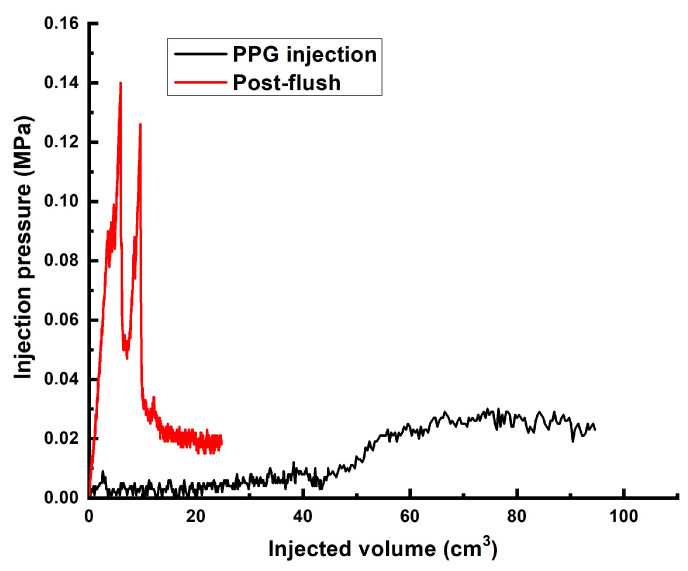
The injection pressure vs. injected volume registered in the course of AAm_95_-SA_5_-impregnated gel injection and brine post-flush. Pore volume of the model—~50 cm^3^.

In the next experiment, 0.075-mm sized AAm_95_-SA_5_ was used to achieve deeper propagation of the particles inside the model. Figure 12 shows the injection pressure values documented during the injection of [0.5 wt.% polymer/0.5 wt.% AAm_95_-SA_5_/0.05 wt.% chromium acetate] gel into the 3 cm diameter and 9 cm long sand pack model. In the course of the PPG-impregnated mature gel injection, the pressure stabilized at around 0.025 MPa (0.27 MPa∙m^−1^), indicating the absence of inlet plugging. Furthermore, the propagation of AAm_95_-SA_5_ through the model is confirmed by their presence in the effluent sample (Figure S5a). The injection was terminated after the PPGs were detected at the outlet of the model.

The post-flush was conducted the day after the gel was injected. The highest pressure recorded during the post-flush was approximately 0.14 MPa (1.5 MPa∙m^−1^), which is about 20 times greater than that in the previous experiment. The movement of the AAm_95_-SA_5_ through the sand pack is evidenced by photos of the sand pack’s inlet and outlet (Figure S5b,c), along with an image of the pack removed from the still pipe after the experiment (Figure S5d).

**Figure 13:**
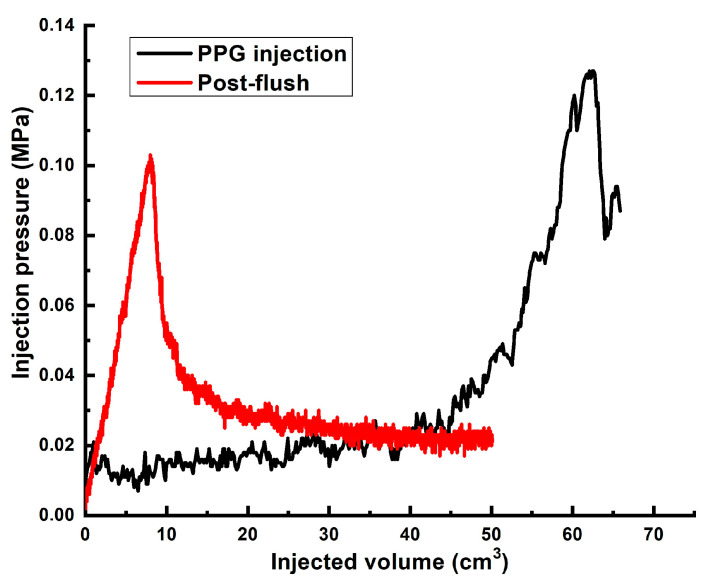
The injection pressure vs. injected volume registered in the course of AAm_95_-APTAC_2.5_-AMPS_2.5_-impregnated gel injection and brine post-flush. Pore volume of the model—~50 cm^3^.

Another experiment was conducted using [0.5 wt.% polymer/0.5 wt.% AAm_95_-APTAC_2.5_-AMPS_2.5_/0.05 wt.% chromium acetate] gel. Figure 13 shows that the injection of the first pore volume of the gel initially exhibited a stable, slow increase in pressure, followed by a sudden exponential surge. This surge can be attributed to the accumulation of PPGs within the high permeability channels, leading to plugging. The maximum pressure recorded the following day during the post-flush reached 0.1 MPa (1.11 MPa∙m^−1^).

Table 4 summarizes the results of the sand pack flooding experiments. For experiments #2–5, the provided residual resistance factor values are approximate due to the limited sensitivity of the pressure measuring device, which could not precisely measure the very low-pressure values (~0.001 MPa at 30 cm^3^∙min^−1^) observed during the pre-flush of the high permeability sand pack models. Higher flow rates were avoided to prevent the disintegration of sand inside the model. The lowest residual resistance factor (RRF) was observed in the first experiment, which can be attributed to the presence of residual oil saturation in the model. Conversely, the highest RRF occurred in the experiment where a gel composed of [0.5 wt.% polymer/0.5 wt.% AAm_95_-APTAC_2.5_-AMPS_2.5_/0.05 wt.% chromium acetate] was injected into the 60 Darcy sand pack model. Considering the evidence of the in-depth propagation of the PPGs used in this formulation and the lack of inlet plugging indicated by the stabilization of the injection pressure, as well as the highest RRF demonstrated by this formulation, #4 formulation was selected as the most promising for field trials.

In the 5.5 Darcy sand pack model, a plain polymer gel reduces permeability by 190 times when residual oil saturation is present in the porous media. However, in practice, the treatment of injection wells with plain polymer gels does not completely shut down injectivity because the multi-Darcy discrete channels consume much of the gellant volume. In the 60 Darcy sand pack model, a polymer gel impregnated with preformed partial gel particles reduced permeability by approximately 8000 times. The stable gel injection pressure gradient of 0.27 MPa*m^−1^, along with the presence of PPGs at the model’s outlet and in the effluent sample, demonstrates that 0.075 mm-sized PPGs at a concentration of 0.5 wt.% are capable of propagating through the 60 Darcy sand pack. The plain polymer gels tested in this study reduced the permeability of the 60 Darcy sand packs by only 730–1950 times.

These results suggest that PPGs may be a superior alternative to plain bulk polymer gels when the permeability of the thief zone ranges in the dozens of Darcies.

## 3. Conclusions

The results of this study demonstrate that polyampholyte hydrogel AAm_95_-APTAC_2.5_-AMPS_2.5_ is more stable under reservoir conditions, while polyelectrolyte hydrogel AAm_95_-SA_5_ loses their swelling capacity in high salinity reservoir conditions. The swelling capacity of both polyelectrolyte and polyampholyte hydrogels is less sensitive to temperature. In water the AAm_95_-SA_5_ and AAm_95_-APTAC_2.5_-AMPS_2.5_ swell up to 56 and 18g/g, respectively. When the ionic strength of the solution increases, the swelling degree of AAm_95_-SA_5_ hydrogel decreases, while the swelling degree of AAm_95_-APTAC_2.5_-AMPS_2.5_ hydrogel remains unchanged. Regarding the effect of pH, the swelling degree of polyelectrolyte gel increases, whereas the swelling degree of polyampholytic hydrogel remains unchanged. Additionally, SEM and BET methods confirmed that both hydrogels are nonporous. With gel strengths ranging between 80 and 90 kPa, no porosity, and approximately equal swelling degrees in saline water (around 10–12 g∙g^−1^), the AAm_95_-SA_5_-based PPG increases the pressure in the bulk model by 140 times, while AAm_95_-APTAC_2.5_-AMPS_2.5_-based PPG increases the pressure by approximately 100 times. Therefore, both systems can be effectively used as PPG.

In a 60 Darcy sand pack, regular HPAM-Cr(III) gel reduced water permeability by 1950 times. Coarse AAm_95_-APTAC_2.5_-AMPS_2.5_ PPGs with high molecular weight polymer gel reduced permeability by only 730 times and could not penetrate the sand pack. However, 0.075 mm AAm_95_-SA_5_ PPGs at 0.5 wt.% reduced water permeability by 8000 times. A stable injection pressure gradient of 0.27 MPa/m and the presence of PPGs in the effluent indicate successful propagation through the sand pack. The main limitation of this work is the low temperature used in the sand pack flooding experiments. Future research will focus on the application of PPGs in high-temperature, fractured carbonate reservoirs with high salinity.

## 4. Materials and Methods

### 4.1. Materials

For synthesis of polyampholyte hydrogel the following chemicals were used: anionic monomer—2-acrylamido-2-methyl-1-propanesulfonic acid sodium salt (AMPS, 50 wt.%); cationic monomer—3-acrylamidopropyltrimethylammonium chloride (APTAC, 75 wt.%); and uncharged monomer—acrylamide (AAm, 99% purity).

The synthesis of polyelectrolyte hydrogel involved the utilization of the following chemicals: acrylamide (AAm, purity of 99%) and acrylic acid. To obtain sodium acrylate (SA) the acrylic acid was fully neutralized by sodium hydroxide.

In both cases, the crosslinker was N,N’-methylenebisacrylamide (MBAA, 99% purity).

Radical polymerization was initiated by ammonium persulfate (APS, 98% purity). All reagents are products of Sigma-Aldrich (USA) and were used without further purification. To improve the mechanical properties of hydrogels a mineral filler—bentonite (BENTOLUX API, Almetyevsk, Tatarstan, Russia)—was used.

For preparation and injection into the oil core model the high molecular weight hydrolyzed polyacrylamide (HPAM) (Sigma Aldrich, St. Louis, MO, USA) crosslinked by chromium acetate (50%) was used for comparative experiments. The ionic strength was adjusted by adding sodium chloride (chemically pure, Reakhim, Moscow, Russia).

Phosphate buffer was used to prepare solutions with desired pH from 2 to 8.

### 4.2. Methods

#### 4.2.1. Synthesis of PPG

Hydrogels of the desired composition were synthesized by free-radical polymerization by mixing the required amount of reagents (the composition of the hydrogels is described in Table 5) and diluting them with distilled water. The initial monomer mixtures (IMM) were stirred for about 2 h and purged with argon for 10 min to remove the dissolved oxygen; then, the required amount of APS was added. The polymerization reaction was carried out in cylindrical vessels with a diameter of 15 mm in a thermostated chamber at 60 °C for 3 h. The synthesized hydrogels were removed from the vessels, cut into discs 5 mm high, and dried to constant weight at room temperature. The dried samples were used for further experiments [49].

#### 4.2.2. FTIR Spectroscopy

FTIR spectra of samples were recorded on a Cary 660 spectrometer (Agilent, Santa Clara, CA, USA) at room temperature in the wavenumber range of 4000–700 cm^−1^. Preliminary the synthesized hydrogels were subjected to freeze-drying for 24 h.

#### 4.2.3. Determination of the Swelling Degree and Swelling Kinetics of Hydrogels in Dependence of Temperature, Ionic Strength, and pH Medium

The swelling kinetics of the hydrogels were measured in distilled water, following the method described in Ref. [42]. The swelling degree (SD) of hydrogels was determined by gravimetric method. A pre-weighed sample of dry hydrogel was immersed in a container with distilled water at a temperature of 24 °C and left to swell. At regular time intervals (5, 10, 15, 30, 60, 120, and 180 min; 1, 2, and 3 days), the hydrogel sample was removed from the water, blotted by filter paper to remove the excess moisture and weighed. The hydrogel was then re-immersed in water again. The SD was calculated using the following Equation (5):

(5) $$SD=\frac{m_{t}-m_{0}}{m_{0}},$$

where SD—swelling degree, g∙g^−1^;$\;m_{t}$—the mass of the sample at a time, g; and $m_{0}$—the mass of dry sample, g.

The temperature-dependent swelling of the hydrogels was performed in the following way. First, the hydrogel sample was weighed and immersed in a container filled with distilled water. This container was then placed in a thermostat set to temperatures of 40 °C, 60 °C and 80 °C. After a 24 h incubation period, the hydrogel was removed from the water, excess moisture was removed by blotting with filter paper, and the samples were weighed again. The degree of swelling was calculated using Equation (5).

The salt-dependent SD was performed as follows: The as-prepared sample was weighed and immersed in sodium chloride solutions with the following different salt concentrations: 1, 10, 25, 50, 75, 100, and 150 g∙L^−1^, and left for a day. After a day, the hydrogel sample was removed from the solution, excess moisture was removed and weighed.

The pH-dependent SD of hydrogels was determined in buffer solutions at pH = 2, 4, 6, 8, 10, and 12 as described above.

#### 4.2.4. Study of Mechanical Properties of Hydrogels

The mechanical properties of the hydrogels were examined using the Texture/Mechanical Analyzer TA.XTplus Stable Micro Systems (Mason Technology, Belfast, UK) using detector with maximal strain 50 kPa. Each hydrogel sample was measured 3 times at room temperature and the results were averaged. The stress–strain diagrams of hydrogels were generated in compression mode. A cylindrical stainless steel probe, P/75, with a diameter of 75 mm was used to press on the hydrogel sample and track the change in compressive force as a function of distance/time. The measurement parameters are as follows: cylindrical probe pre-test speed of 1 mm∙s^−1^, test speed of 0.5 mm∙s^−1^, release force of 0.2 g, and remote target mode with a distance of 1.5 mm. The tension value (force per unit area) was calculated from the maximum force value (Equation (6)), as follows:

(6) $$Stress=\frac{Force\;(g)}{Area{(mm}^{2})}$$

Since the stress curve is proportional to the strain, the slope is Young’s modulus. This means that the gel material only deforms elastically in this area [56].

#### 4.2.5. Study of Surface Morphology of Hydrogels

To study the surface morphology, the hydrogel samples were analyzed using the low-vacuum analytical scanning electron microscope JSM-6390 LV (JEOL, Akishima, Japan) (SEM). Sample preparation was performed using a JEE-420 vacuum sputtering unit (JEOL, Akishima, Japan), with carbon rods and a JEOL DATUM (Akishima, Japan) to form a thin conductive carbon film.

#### 4.2.6. Study of the Porosity of Hydrogels

The specific surface area of hydrogels was determined from the krypton Krad sorption isotherms at −196 °C. The samples (~0.40–0.60 g) were first degassed using a Micromeritics FlowPrep 060-type at 80 °C for overnight at static condition before transferring to the Micromeritics 3Flex 3500-type instrument (USA) and then continued under vacuum at 80 °C for a further 4 h. The Brunauer–Emmett–Teller (BET) method within a Kr relative pressure range of 0.05–0.30 was used to calculate the specific area (S_BET_).

#### 4.2.7. Sand Pack Flooding Experiments

In the first experiment 4.3 cm diameter and 8.6 cm length sand pack model with no discrete high permeability channels was used. Pore volume and permeability of this model to brine at 100% water saturation were equal to ~5.5 and 50 Darcy, respectively, whereas permeability to brine at residual oil saturation was assessed to be 165 mD.

The experiment on this model was conducted in the following sequence:

- Reservoir unconsolidated oil-wet sand was packed into a steel cylinder with a diameter of 4.3 cm and a length of 8.6 cm;
- The permeability of the model was determined using 26.6 g·L^−1^ brine from the Karazhanbas oilfield. The brine’s composition is listed in Table 6;
- The model was saturated with crude oil at 1 cm³·min^−1^ and 25 °C. The viscosity 796.39 The cP and density of the oil were equal to 796 cP and 0.93 g·cm^-^³ (20° API), respectively;
- Oil was displaced using 26.6 g·L^−1^ brine at a rate of 1 cm³·min^−1^ and a temperature of 25 °C to establish the initial conditions;
- Freshly prepared [0.5 wt.% 6–7 mln Da and 5% hydrolysis degree HPAM/0.5 wt.% chromium acetate] gellant was injected into the model at a rate of 1 cm^3^/min and a temperature of 25 °C. The gellant was prepared by using fresh low salinity brine;
- The model was left for 3 days to allow the composition to gel;
- After 3 days, the model was subjected to brine injection in the direction opposite to the gellant injection.

Other experiments were conducted by using sand pack models with discrete high-permeability channels. In order to make these sand pack models, reservoir sand from a depth of 425.88 m was used as received with the resident oil/water in place. Since the partially formed gel particles are not likely to penetrate small pores, 1–2.6 cm sized sand particles were used to fill the sand pack in order to create a multi-Darcy permeability model of porous media (Figure S7). The sand pack diameter and length were equal to 3 and 9 cm, respectively. The pore volume and permeability of these sand pack models were equal to ~50 cm^3^ and >60 Darcy, respectively.

In general, the experiments on >60 Darcy sand pack models were conducted in the following sequence:

(1) Vacuuming the sand pack;
(2) Injecting 26.6 g∙L^−1^ brine at 5 cm^3^∙min^−1^ (Figure S8). Calculating pore volume and porosity;
(3) Gel injection at 1 cm^3^∙min^−1^;
(4) Post-flushing with 26.6 g∙L^−1^ brine at 1 cm^3^∙min^−1^.

**Figure 14:**
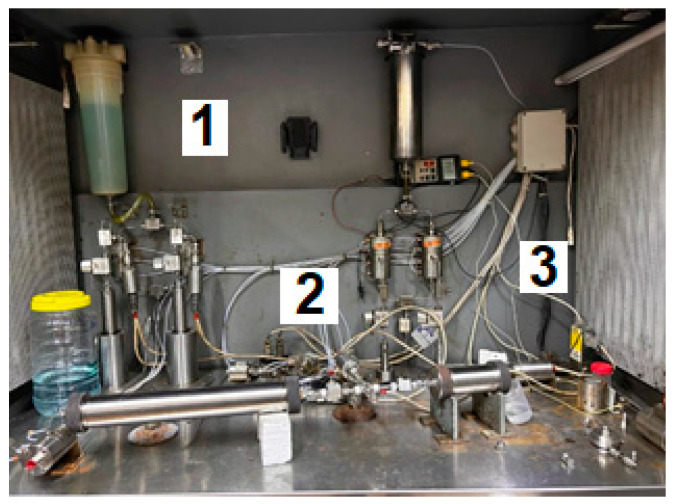
Core flooding setup (1—high pressure pump, 2—accumulator, and 3—sand pack).

All sand pack flooding tests were conducted at 25 °C, which is representative of the actual reservoir temperature at the Karazhanbas oil field. The core flooding setup shown in Figure 14 was unitized. The core flooding setup shown in Figure 14 was unitized. High-pressure pumps were used to inject distilled water into the accumulator to push on the piston and displace brine or gel into the sand pack. One pressure measuring device was used to monitor the injection pressure at the inlet of the sand pack model. Of importance to note is that the inner diameter of the injection line was equal to 4 mm.

#### 4.2.8. Preparation of PPG-Impregnated Gel Samples for the Flooding Experiments

To prevent the gravity segregation of the preformed gel particles inside of the accumulator, PPG particles were used as a suspension in HPAM gel, as follows:

0.5 wt.% of dry HPAM powder was added to 26.6 g∙L^−1^ brine and stirred for 1 h at 300 rpm using an impeller overhead stirrer.

0.5 wt.% of PPG particles were added to the solution and stirred for additional 3 h. After stirring for 4 h, 0.05% of chromium acetate was added to induce gelation (i.e., viscosity increase).

The samples were kept at room temperature and periodically agitated to keep the PPG particles evenly suspended throughout the volume of the solution. As a result, the increase in viscosity that occurred overnight was enough to prevent gravity segregation of the PPGs. In real field conditions, PPGs can be dispersed in brine and injected directly, with dynamic wellbore flow preventing segregation.

## Figures and Tables

**Table 1:** Examples of different PPGs applied for enhanced oil recovery.

Polymer	Key Results	Disadvantages/Problems	Reference
PAM and chitosan	Swelling capacity and storage modulus Swelling ratios vary between 5 and 107 g/g in DIW and 7 and 21 g/g in HSW. Higher temperatures and fresh water boost swelling, while more chitosan and neutral pH increase the storage modulus.	Chitosan is not soluble in water only in acidic media Mechanical properties of synthesized hydrogels is approximately weak Noncovalent interactions leads to degradation of gels under high salinity	[4]
Poly (acrylamide-co-acrylic acid)	Mechanical and swelling abilities Increasing acrylic acid boosts PPG swelling but weakens the network, while more crosslinker reduces swelling and strengthens the gel	What is crosslinker in these study is unclear Conventional PPGs expand too quickly in reservoir pores, making it difficult to effectively control deep profiles. Slow-swelling PPGs are easier to inject into deeper reservoirs, significantly improving plugging efficiency.	[33]
Agar/PAM	- Thermogravimetric analysis - Swelling measurements - Particle size analysis - Rheological properties - Swelling ratio: 8.4 to 19.2 g/g - Stable up to 130 °C - Storage modulus: 10 to 32 kPa - Effective in sealing open fractures in sandstone cores with agar-based material - Agar-based PPG shows good plugging efficiency with a water breakthrough pressure of approximately 108.9 kPa (778 kPa/m) in open fracture models.	High porosity of gels can lead to reduced plugging efficiency and allow free water transport through the pores, especially since swelling decreases and pore diameter increases in saline water.	[34]
Poly(acrylamide-acrylic acid-2-methylpropanesulfonate) (AM-AA-AMPS) and aluminum (III) ions	The swelling behavior of these hydrogels and their interaction with rock samples were investigated. - Polymer film formation on rock surfaces can alter wettability. - Contact angle measurements showed that PPGs can change rock wettability, particularly in carbonate samples, from oil-wet to water-wet	The anionic nature of the polymer matrix significantly influences the swelling degree of hydrogels, as it is strongly affected by the ionic strength of the solvent and the pH.	[35]
Alkaline surfactant polymer (ASP)	- Viscosity and stability assessment—Rheological properties - Oil displacement experiments using a 3D physical model and oil saturation monitoring device - The PPG/ASP system’s viscosity was 30% higher than the single polymer solution - The system’s viscosity retention rate over 30 days was 89.83%, significantly higher than the polymer system. - -Stronger shear resistance in the PPG/ASP system. - Oil recovery increased by 14.6%, with a recovery rate of 28.6% in low permeability layers	The necessity of using a surfactant modifier Reduction in swelling with increasing ionic strength of the solution	[36]
4–6 Alkylene bisacrylamide and AAm	- Viscosity performance - Stability assessment - Rheological properties - Oil displacement experiments with 3D physical model and oil saturation monitoring device - The heterogeneous composite system’s viscosity is 30% higher than that of a single polymer solution. - It retains 90% of its viscosity over 30 days, 17% higher than the polymer system. - - It increased oil recovery by 13.56% after polymer flooding	Reduction in swelling degree with increasing salt concentration	[37]

**Table 2:** Constants of the Rittger–Peppas equation.

Sample	$S_{e}$	A	N	R^2^	Main Process
AAm_95_-SA_5_	28.97	0.13	0.91	0.99	Relaxation and diffusion
AAm_95_-APTAC_2.5_-AMPS_2.5_	12.27	0.12	0.71	0.99	Relaxation and diffusion

**Table 3:** Data on the *SD* of hydrogels.

Sample	$S_{e}$	$k_{1}$	$k_{2}$	R^2^	Main Process
AAm_95_-SA_5_	28.98	0.002	−0.023	0.99	Relaxation and diffusion
AAm_95_-APTAC_2.5_-AMPS_2.5_	12.27	0.005	0.008	0.99	Relaxation and diffusion

**Table 4:** The results of the sand pack flooding experiments.

#	Sand Pack Permeability to Brine, DARCY	Diameter, cm/Length, cm of the Model	Gellant/Gel Recipe	Injected Volume of Gellant/gel	Maximal Gellant/gel Injection Pressure, MPa	Maximal Post-Flush Injection Pressure, MPa	Assessed RRF
1	0.165 *	4.3 cm/8.6 cm	Gellant [0.5 wt.% 6–7 mln Da and 5% hydroyzed HPAM/0.5 wt.% chromium acetate]	3 PVs	0.088	7	190
2	~60	4.3 cm/8.6 cm	Gellant [0.5 wt% 6–7 mln Da and 5% hydrolyzed HPAM/0.2 wt.% chromium acetate]	1 PV	Too low to be detected	0.016	~1950
3	~60	4.3 cm/8.6 cm	Gel [0.5 wt.% 13–18 mln Da and 16.5% hydrolyzed HPAM/0.5 wt.% coarse (1–2.5 mm) PPG AAm_95_-APTAC_2.5_-AMPS_2.5_/0.05 wt.% chromium acetate]	1 PV	0.042	0.006	~731
4	~60	3 cm/9 cm	Gel [0.5 wt.% 13–18 mln Da and 16.5% hydrolyzed HPAM/0.5 wt.% fine PPG AAm_95_-SA_5_/0.05 wt.% chromium acetate]	2 PVs **	0.028	0.14	~8000
5	~60	3 cm/9 cm	Gel [0.5 wt.% 13–18 mln Da and 16.5% hydrolyzed HPAM/0.5 wt.% fine PPG AAm_95_-APTAC_2.5_-AMPS_2.5_/0.05 wt.% chromium acetate]	1.32 **	0.126	0.1	~5714

* The permeability to brine at 100% water saturation—5.5 Darcy. ** The injection was continued until PPGs were detected at the outlet of the model.

**Table 5:** Ratio of monomers in the initial monomer mixture for the production of hydrogels.

Component	AAm-SA		AAm-APTAC-AMPS	
	mol.%	m, mg	mol.%	m, mg
AAm	95	444.2	95	733.9
SA	5	5	-	-
APTAC	-	-	2.5	106.8
AMPS	-	-	2.5	141.6
MBAA	1	10.7	1	17.7
Bentonite	5	0.5	5	0.5
Water	90	9	85	8.5
Monomer concentration, %	5		10	

**Table 6:** Brine composition.

Parameter	Value
TDS, g/L	26.6
K^+^, mg/L	70.94
Ca^2+^, mg/L	2
Mg^2+^, mg/L	12.2
Na^+^, mg/L	12,532
SO_4_^2−^, mg/L	40
Cl^−^, mg/L	17,373.9

## Data Availability

The data presented in this study are openly available in article.

## Supplementary Materials

The following supporting information can be downloaded at: https://www.mdpi.com/article/10.3390/gels10090562/s1, Figure S1: Stress-strain curves of hydrogels; Figure S2: Dependence of Young’s modulus of hydrogels on their composition; Figure S3: The injection pressure vs injected volume in the course of the injection of 0.5 wt.% 6-7 mln Da HPAM/0.5 wt.% chromium acetate gelling solution into 5.5 Darcy sand pack model; Figure S4: (a) Inlet face of the sand pack model after the experiment; (b) coarse sand and polymer gel extracted from the model after the experiment; Figure S5: (A) Effluent sample collected at the outlet of the sand pack model during the injection of [0.5 wt.% polymer/0.5 wt.% AAm95-SA5/0.05 wt.% chromium acetate] gel; (B) inlet; (C) outlet of the sand pack; (D) sand pack after the experiment; Figure S6: (A) Effluent sample collected at the outlet of the sand pack model during the injection of [0.5 wt.% polymer/0.5 wt.% AAm95-APTAC2.5-AMPS2.5 0.05 wt.% chromium acetate] gel; (B) inlet; (C) outlet of the sand pack; (D) sand pack after the experiment; Figure S7: Preparation of sand for the flooding experiment; Figure S8: Saturating sand pack model with 26.6 g∙L-1 brine.

## Abbreviations

Aam	Acrylamide
AMPS	2-Acrylamido-2-methyl-1-propanesulfonic acid sodium salt
APTAC	3-Acrylamidopropyltrimethylammonium chloride
DSC	Differential scanning calorimetry
EOR	Enhanced oil recovery
GTP	Group transfer polymerization
HPAM	Hydrolyzed poly(acrylamide)
IMM	Initial monomer mixtures
MBAA	N,N’-methylenebisacrylamide
PPG	Preformed particle gels
SA	Sodium acrylate
SD	Swelling degree
TDS	Total dissolved solids
TGA	Thermogravimetric analysis
