# Supplementary material for: Preparation and Characterization of Preformed Polyelectrolyte and Polyampholyte Gel Particles for Plugging of High-Permeability Porous Media
Source: Gels. 2024 Aug 29;10(9):562. doi: 10.3390/gels10090562 (PMC11430932; doi:10.3390/gels10090562)
Supplement: Supplementary file 1 [file gels-10-00562-s001.zip › gels-3145776-supplementary.pdf]

Supplementary materials

Preparation and characterization of preformed polyelectrolyte and polyampholyte gel particles for plugging of high-permeability porous media

G.T. Yelemessova[2], I.Sh. Gussenov[1,3], A.Ye. Ayazbayeva[1], A.V. Shakhvorostov[1*], L.K. Orazzhanova[2], A.N. Klivenko[1,2], S.E. Kudaibergenov[1]

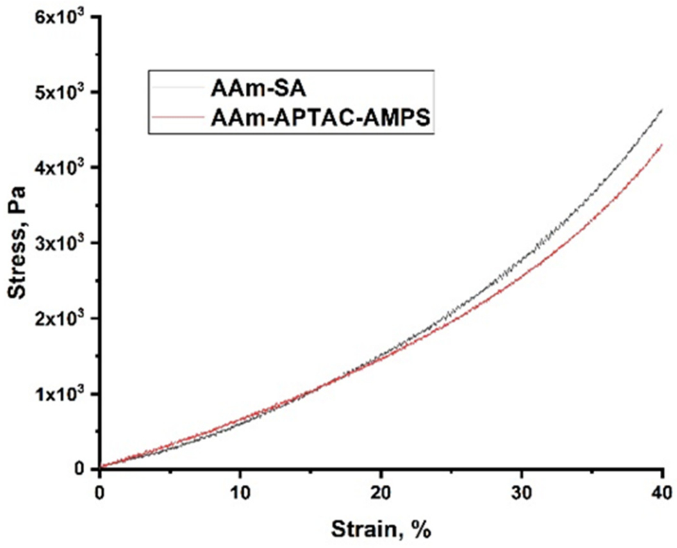

Figure S1. Stress-strain curves of hydrogels

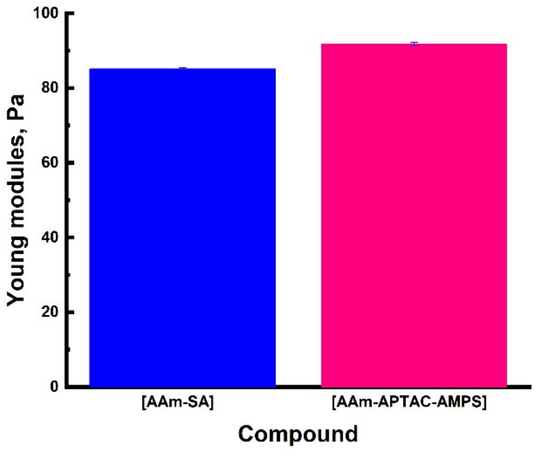

Figure S2. Dependence of Young's modulus of hydrogels on their composition

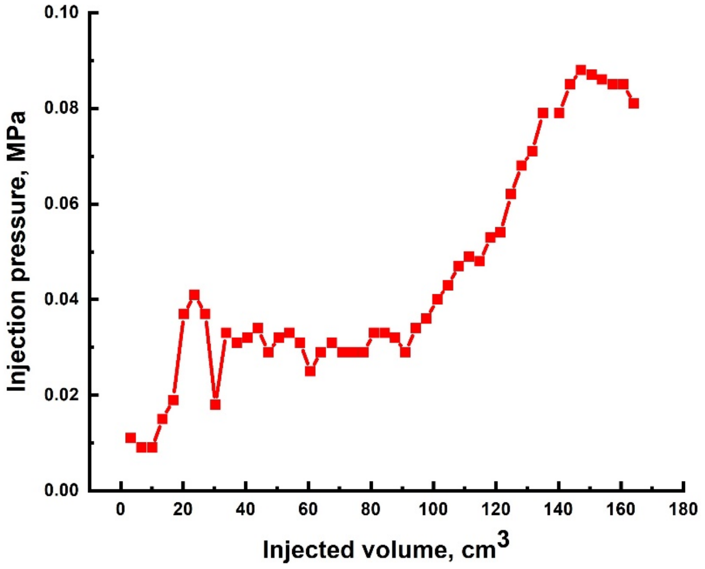

Figure S3. The injection pressure *vs* injected volume in the course of the injection of 0.5 wt.% 6-7 mln Da HPAM/0.5 wt.% chromium acetate gelling solution into 5.5 Darcy sand pack model

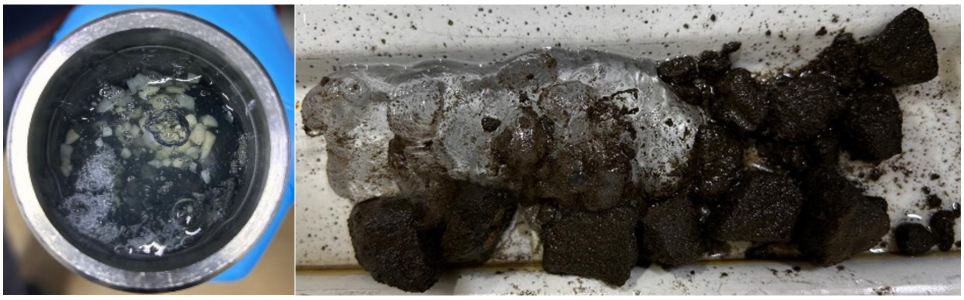

a) b)

Figure S4. a) Inlet face of the sand pack model after the experiment; b) coarse sand and polymer gel extracted from the model after the experiment

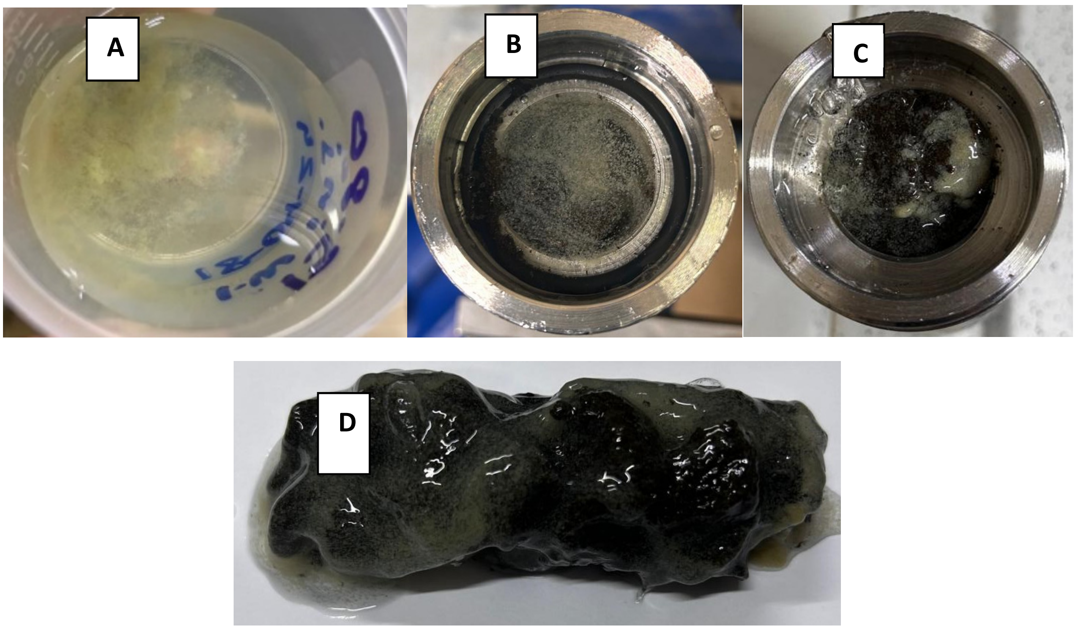

Figure S5. A) Effluent sample collected at the outlet of the sand pack model during the injection of [0.5 wt.% polymer/0.5 wt.% AAm_{95}-SA_5/0.05 wt.% chromium acetate] gel; B) inlet; C) outlet of the sand pack; D) sand pack after the experiment.

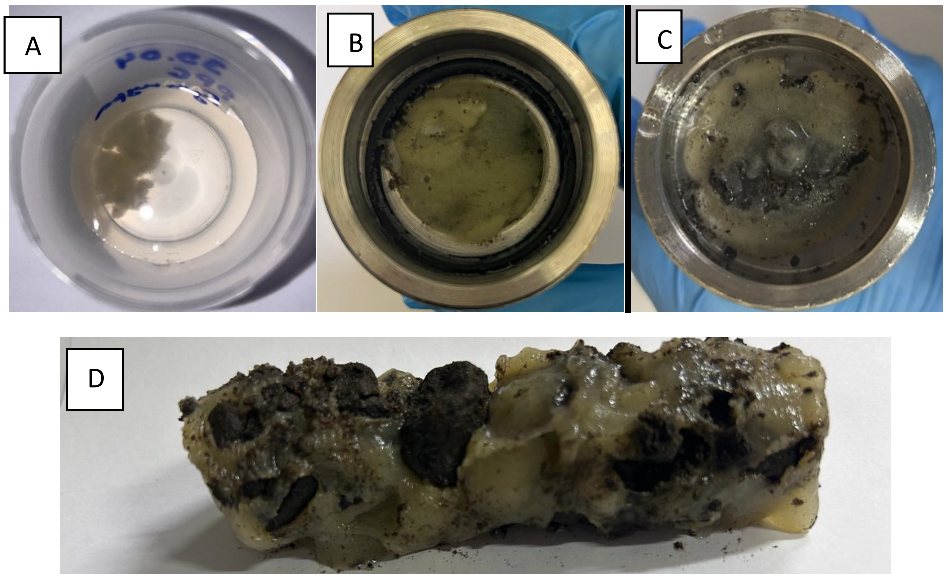

Figure S6. A) Effluent sample collected at the outlet of the sand pack model during the injection of [0.5 wt.% polymer/0.5 wt.% AAm$_{95}$-APTAC$_{2.5}$-AMPS$_{2.5}$ 0.05 wt.% chromium acetate] gel; B) inlet; C) outlet of the sand pack; D) sand pack after the experiment.

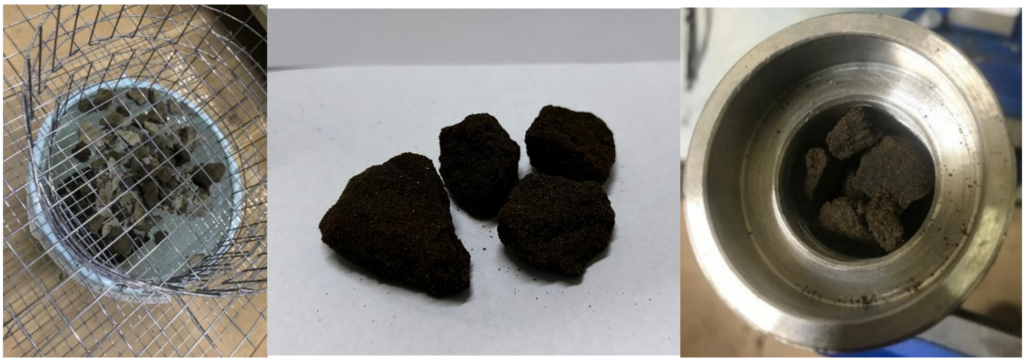

Figure S7. Preparation of sand for the flooding experiment.

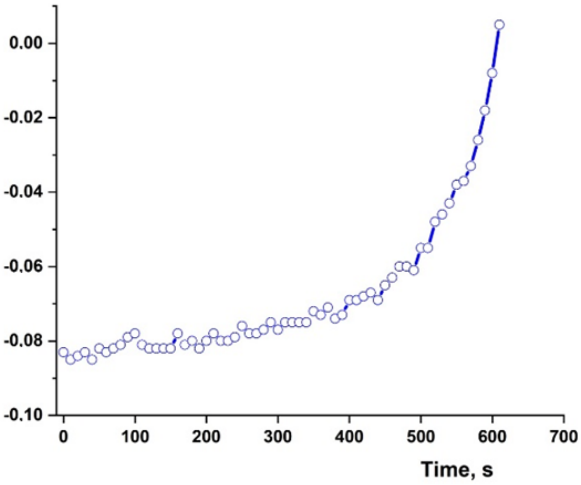

Figure S8. Saturating sand pack model with 26.6 g·L^{-1} brine.
